# The Politics of Covid Vaccine Hesitancy and Opposition

**DOI:** 10.1111/1467-923X.13134

**Published:** 2022-04-21

**Authors:** Tom Sorell, Jethro Butler

**Keywords:** anti‐vax, vaccine hesitancy, populism, conspiracy theories, social media

## Abstract

Opposition to vaccines is not a new phenomenon, but positions once associated with traditional religious or conservative stances have given way to some highly disparate views that transcend traditional left/right/religious divisions. This article reviews recent literature showing how social media has contributed to the spread of conspiracy theories around Covid‐19 and mass vaccination programmes. The narratives discussed are principally those of the right and the religious right.

OPPOSITION TO vaccines is not new.^1^ However, positions once associated with traditional religious or conservative viewpoints have given way to some highly disparate views: ultra‐right‐wing racist and antisemitic conspiracy theories; anti‐globalist conspiracy movements of both the left and right; suspicion of big pharma; left‐wing and libertarian suspicion of routine government overreach; and 5G conspiracy theories that draw adherents from both the right and from the ecological movement.

There is more than one way to doubt the benefits of a vaccine programme. For example, to be vaccine hesitant is not necessarily to be anti‐vax. It is possible for a layperson to believe that trialled vaccines in general are effective and yet to have doubts as to whether a particular, newly introduced, vaccine reduces the risk of serious illness or reduces infection. Vaccine hesitancy along these lines will be disregarded in what follows. We also set to one side vaccine hesitancy influenced by conventional scientific disagreement about the likely evolution of an infectious disease.^2^ We concentrate instead on outright opposition to vaccines and, what is more, opposition to vaccines associated with opposition to power structures that vaccine programmes are seen to represent.^3^ Our review extends beyond recent politics literature to papers in social psychology and medical anthropology. Vaccine scepticism is in some respects a reaction against elites, including experts, when the lives of a whole people are affected. It is also at home among believers in conspiracy theories. We have selected very recent and often multidisciplinary papers to reflect the increasing use of hybrid theoretical approaches to these phenomena.

The fact that the pandemic has been global and has brought into play global institutions such as the World Health Organization (WHO), has been seen by some anti‐vaxxers as a sign of the operation of a powerful globalising cabal intent on dismantling the current system of nation states and instituting a new world order. Before the pandemic, the United Nations was seen by Trump and some of his followers as a channel for an objectionable globalising agenda.^4^ After 2020, WHO was seen in the same light. Just how many anti‐vaxxers outside America believe in this sort of creeping transnational power grab is unclear. But even where anti‐vaxxers do not suspect the emergence of a new and sinister world order, they have sometimes associated vaccination programmes with disproportionate interference by governments in the activities of schools, churches and small businesses.

Right‐wing narratives predominate. Sturm and Albrecht point to the way in which Covid has mobilised religious promoters of an imminent apocalypse and secular believers in conspiracy theories.^5^ In the US particularly, they identify a trend among grassroots evangelicals of associating emerging disease, especially disease constructed as coming from abroad, with a biblically forecast end of the world. The American millenialist narrative has spread, Sturm and Albrecht suggest, to Europe, and they cite evidence from Belfast and Berlin: ‘Millennial and conspiracist narratives … can act as sustainable and powerful political resources for decades as long as they are constantly adjusted to reflect contemporary discourses, fears and crises.’ Interestingly, Sturm and Albrecht establish the prescience of Richard Hofstader's 1964 essay, ‘The American paranoid style’, which captures uncannily well the tendencies we are currently experiencing, notwithstanding the fact that it predates social media by many decades. They also demonstrate the compatibility of a religious ‘end times’ narrative with predictions in 2020 from the notorious extreme right secular conspiracist, Alex Jones, of a ‘one world’ communist government.

Although anti‐vax positions are predominantly right‐wing, Sturm and Albrecht present a compelling medical‐anthropological analysis of these conspiracy movements and how their narratives cut across left, right and religious categories. Conspiracy theories have drawn together both the left and right of the political spectrum on lockdown and vaccine scepticism.^6^ Left‐wing anti‐vax movements are less conspicuous, but they exist. An example not discussed by Sturm and Albrecht is a post‐Covid narrative promoted by Edward Snowden whereby Covid surveillance apps are a continuation, and maybe an intensification, of post 9/11 government surveillance associated with counter‐terrorism.^7^

Johnson and Velasquez show how, in the US, otherwise apolitical parents using online forums during Covid school shutdowns were put in touch with a host of conspiracy theories about Covid‐19 and vaccines in Facebook groups.^8^ The connecting link was vaccine‐sceptical posts placed on online forums by supporters of ‘alternative health’. Anti‐vax sentiment along these lines grows out of a general suspicion of artificial or manufactured aids to health and wellbeing, and a belief in ‘natural’ cures and therapies. Long before Covid, New Age views affected take‐up of the MMR vaccine.^9^ Although New Age anti‐vaxxers can sometimes share with other opponents of vaccines a belief in the threat posed by big pharma—highly profitable and politically influential developers of drugs—they are not necessarily ideologically close to anti‐vaxxers who see vaccine mandates as one instance among others of the exercise of increasingly centralised state power. But, in a world of loosely connected interest groups so familiar from social media, extreme views can catch on even with moderates: parents angry at the closure of schools can be receptive to a conspiracist framing of school lockdowns while being unreceptive to conspiracists and conspiracies in general.

Nathalie Van Raemdonck presents an analysis of the ways in which people are drawn into radical narratives online and how this is a particular problem with the way Facebook has designed the rules for groups: unlike Reddit which, according to Raemdonck, is designed to facilitate more open debate, Facebook groups, through their privacy settings and moderation procedures, operate as closed echo chambers that serve to reinforce the views of the members of the group. As a result, even though Reddit can and does host views and opinions that are hateful and repugnant, those views are always open to challenge, whereas the moderators of Facebook groups operate according to rules that oblige them to excise dissenting comments.^10^

Weinberg and Dawson present a sociological analysis of the mechanisms by which people with disparate interests and commitments are drawn together around common issues and grievances through the use of grand identity‐forming narratives.^11^ The narratives in question radically revise the connotations of the phrase ‘ordinary Americans’, for instance, harking back to an idealised and mythical past, and linking common but vague commitments to freedom and patriotism with very specific issues like gun ownership and resistance to vaccinations. In the US, gun ownership is constitutionally protected and often interpreted by its supporters as a protection against the permanent possibility of tyranny. Lockdowns of schools and businesses during the Covid pandemic were viewed by the same people as incipient tyranny, and this view was attractive to some parents seeking to frame their own frustration with the closure of schools.

Probably the most comprehensive survey of American right‐wing conspiracy theories connected with Covid in general is in Bodner, et al., *COVID‐19 Conspiracy Theories: QAnon, 5G, the New World Order and Other Viral Ideas*.^12^ This book‐length investigation does not confine itself to vaccine hesitancy or the anti‐vax movement, but considers questions about: the geographical and biological origin of the virus; an allegedly long established willingness of the American health establishment to experiment on black people; the connection between Covid policy and a so‐called power elite called the ‘deep state’; the alleged personal paedophilic tendencies of members of that elite supposedly operating from a Washington D.C. pizza parlour (Pizzagate); and Covid as an indicator of the arrival of the biblical end times. The connection between 5G and Covid is through the false belief that vaccine injections implant microchips capable of identifying individuals and controlling behaviour through mobile phone masts that are omnipresent in developed countries. Another variation on the belief that Covid and 5G are linked is to the effect that electromagnetic waves from transmitter towers have caused the virus.^13^ Bill Gates specifically is associated with the microchip version of the conspiracy theory, and he is anyway regarded by believers in Covid conspiracy theories as a member of the objectionable power elite in the US.^14^

Bodner, et al. recognise that the anti‐vax movement long predates Covid. Chapter 5 of their book takes the reader through vaccine scepticism from well before the nineteenth century. Organised anti‐vaxxers such as the Leicester Anti‐Vaccination League appeared in England after the promulgation of the 1840 Vaccine Act. The pamphlets of these early anti‐vax groups seized on the alleged ambitions of the state to control people's bodies and unduly limit their freedoms. Reporting the results of medical folklorist Andrea Kitta, Bodner, et al. summarise the doubts of twenty‐first century opponents of vaccines in four points: (1) vaccines cause disease, for example, autism or attention deficit disorder; (2) vaccines are for profit; (3) vaccines do not improve immunity; and (4) vaccines are not natural.

For some specific groups, American blacks in particular, vaccines are offered by the same government medical agency (the Centers for Disease Control) that organised the infamous Tuskegee Study (1932–1972). In that experiment, 400 black Americans were used as a control group of untreated sufferers of syphilis when treatment was in fact available. These research subjects were not informed properly about the purpose of the experiment and were thus involved in the experiment without consenting to it. This scandal is the foundation of much minority distrust of Covid vaccines in the US.

Outside the US, Bodner, et al. report many proposals to test vaccines first in Africa. A possible effect in Africa of the perception that it is being used as a vaccine testbed is the association of vaccination with sterility. In 2010, a news story circulated that a Gates Foundation‐funded initiative had used a contraceptive called Depo‐Provera on unknowing villagers in Navrongo, a rural region of Ghana. This story was denounced (but not effectively discredited) by Ghanaian health professionals.

As for big pharma, this branch of business is easily associated with the deep state, the alliance of industry and government elites that is allegedly working behind the scenes to control American policy in every sector. Interestingly, the commonplace right‐wing belief in a nefarious deep state is not necessarily accepted by the relatively well‐educated anti‐vax movement. What is decisive for them, according to a June 2019 Harris Poll of 2000 US adults, is different: ‘45 per cent had doubts about vaccine safety. The top three reasons were based on online articles (16%), past secrets/wrongdoing by the pharmaceutical industry (16%) and information from medical experts (12%).’^15^

Much current anti‐vaxxer sentiment originates in the US. How prevalent is it in Europe? Research from the Horizon Europe project, RECoVER (Rapid European SARS‐CoV‐2 Emergency Research response), includes an online survey conducted in December 2020 of 7000 respondents in seven European countries: Belgium, France, Germany, Italy, Spain, Sweden and Ukraine.^16^ The survey was carried out by IPSOS and implemented with panels of 1000 respondents aged 18–65 in each country. The respondent sample was stratified by gender, age, and geographical region. The survey showed that respondents in France were the most vaccine hesitant: only 44 per cent of those surveyed said they would be willing to use a vaccine certified as safe by a scientific paper, with willingness increasing only to 61 per cent in the most vaccine confident country, Ukraine. RECoVER does not report that vaccine hesitancy in these cases is affected by conspiracy theories, and the fact that many people responded positively to the question of whether they would use a vaccine certified by scientists as safe shows that respondents do not fit the profile of groups discussed by Bodner, et al.

There is nevertheless evidence in Europe of an association between, on the one hand, vaccine hesitancy and vaccine opposition and, on the other hand, populism. Recio‐Román, et al. show that in the whole array of EU member states, vaccine hesitancy or opposition can be interpreted as a sign of a general populist opposition to elites, where elites are constituted by politicians, intellectuals, the media and ‘experts’, including health care specialists.^17^ Popular opposition to public health advice is partly a matter of doubting the competence of the relevant experts or sensing in their guidance an agenda of control of ‘the people’. Or it can be a matter of distrusting state institutions such as health services. Recio‐Román, et al. attribute vaccine opposition or hesitancy to the belief that vaccines are useless, and to the connection between populist ideas and distrust. Distrust was high in countries with a Soviet past; it was lowest in the Netherlands and Scandinavia.

The association between populism and vaccine hesitancy in Europe is also borne out by Jonathan Kennedy, who shows a strong correlation between the belief that ‘vaccines are not important for children to have’ and electoral support for populist political parties.^18^ The strength of this correlation is highest in Italy—among supporters of the Five Star movement. At the other end of the spectrum of fourteen countries studied is Portugal; England and France are closer to the middle.

Freeman et al have published similar results. Strong vaccine scepticism in England, they found, was felt by 10 per cent of the population.^19^ What is more, there is appreciable endorsement of conspiracy beliefs about coronavirus. Such ideas do not appear to be confined to the fringes. Conspiracy beliefs connect to other forms of mistrust and are associated with less compliance with government guidelines and greater unwillingness to take up future tests and treatment.

To sum up, although anti‐vax views and the conspiracy theories that swirl around the Covid‐19 vaccination programme are predominantly narratives of the political right and the religious right, anti‐vax views (and views that are generally sceptical of lockdown and other measures associated with combatting the virus) now extend across the political spectrum. Opposition to vaccines and hesitancy to use them track a breakdown in trust between many citizens and traditional political authorities. To the extent that conspiracy theories about vaccines and scepticism about medical and scientific authority can amplify distrust and disaffection with political authority, this is a trend with worrying public health and political implications.

## Notes

^1^
See for example, D. Porter and R. Porter, ‘The politics of prevention: anti‐vaccinationism and public health in nineteenth‐century England’, *Medical History*, vol. 32, 1988, pp.231–52; https://www.ncbi.nlm.nih.gov/pmc/articles/PMC1139881/pdf/medhist00062‐0007.pdf (accessed 9 March 2022).

^2^
M. Dahlquist and H. D. Kugelberg, ‘Public justification and expert disagreement over non‐pharmaceutical interventions for the COVID‐19 pandemic’, *Journal of Medical Ethics*, 2021; 10.1136/medethics‐2021‐107671 (accessed 11 April 2022).

^3^
D. A. Stecula, O. Kuru, D. Albarracin and K. Hall Jamieson, ‘Policy views and negative beliefs about vaccines in the United States’, *American Journal of Public Health*, vol. 110, 2019, pp.1561–1563; https://doi.org/10.2105/AJPH.2020.305828 (accessed 11 April 2022).

^4^
D. Bosco, ‘We've been here before: the durability of miulti‐lateralism’, *Journal of International Affairs*, vol. 70, no. 2, 2017, pp. 9–15.

^5^
T. Sturm and T. Albrecht, ‘Constituent Covid‐19 apocalypses: contagious conspiracism, 5G, and viral vaccinations’, *Anthropological Medicine*, vol. 28, no. 1, 2021, pp.122–39; https://pubmed.ncbi.nlm.nih.gov/33233926/ (accessed 9 March 2022).

^6^
Sturm and Albrecht, ‘Constituent Covid‐19 apocalypses’; R. Blaskiewicz, ‘The big pharma conspiracy theory’, *Medical Writing*, vol. 22, no. 4, 2013, pp. 259–61 https://journal.emwa.org/good‐pharma/the‐big‐pharma‐conspiracy‐theory/ (accessed 9 March 2022).

^
**7**
^
I. A. Hamilton, ‘Edward Snowden says COVID‐19 could give governments invasive new data‐collection powers that could last long after the pandemic’, *Insider*, 27 March 2020; https://www.businessinsider.com/edward‐snowden‐coronavirus‐surveillance‐new‐powers‐2020‐3?r=US&IR=T (accessed 11 April 2022).

^8^
N. F. Johnson, N. Velasquez, et al., ‘Mainstreaming of conspiracy theories and misinformation’, *arXiv preprint arXiv*; 2021; https://arxiv.org/ftp/arxiv/papers/2102/2102.02382.pdf (accessed 9 March 2022). See also, S. A. Baker, ‘Alt. health influencers: how wellness culture and web culture have been weaponised to promote conspiracy theories and far‐right extremism during the COVID‐19 pandemic’, *European Journal of Cultural Studies*, vol. 25, no. 1, 2022, pp. 3–24; https://journals.sagepub.com/doi/full/10.1177/13675494211062623 (accessed 9 March 2022).

^9^
See T. Sorell, ‘Parental choice and expert knowledge in the debate about MMR and autism’, in *Ethics, Prevention, and Public Health*, A. Dawson and M. Verweij, eds., Oxford, Oxford University Press, 2007, pp. 97–109.

^10^
N. Van Raemdonck, ‘The echo chamber of anti‐vaccination conspiracies: mechanisms of radicalization on Facebook and Reddit’, *SSRN*, Institute for Policy, Advocacy and Governance (IPAG) Knowledge Series, 2019; https://ssrn.com/abstract=3510196 (accessed 9 March 2022).

^11^
D. Weinberg and J. Dawson, ‘From anti‐vaxxer moms to militia men: influence operations, narrative weaponization and the fracturing of American identity’, *Brookings Institute*, October 2021; https://www.brookings.edu/wp‐content/uploads/2021/10/FP_20211108_influencer_operations_dawson_weinberg_v2.pdf (accessed 9 March 2022).

^12^
J. Bodner, et al., *COVID‐19 Conspiracy Theories: QAnon, 5G, the New World Order and Other Viral Ideas*, Jefferson SC, McFarland, 2020.

^13^
D. Jolley and J. L. Paterson, ‘Pylons ablaze: examining the role of 5G COVID‐19 conspiracy beliefs and support for violence’, *British Journal of Social Psychology*, vol. 59, no. 3, 2020, pp. 628–640; https://bpspsychub.onlinelibrary.wiley.com/doi/10.1111/bjso.12394 (accessed 11 April 2022).

^14^
E. Thomas and A. Zhang, ‘ID2020, Bill Gates and the mark of the beast: how Covid‐19 catalyses existing online conspiracy movements’, *Australian Strategic Policy Institute*, 2020; http://ad-aspi.s3.amazonaws.com/2020-06/ID2020%2C%20Bill%20Gates%20and%20the%20Mark%20of%20the%20Beast_%20how%20Covid-19%20catalyses%20existing%20online%20conspiracy%20movements.pdf (accessed 11 April 2022).

^15^
Bodner, et al., *COVID‐19 Conspiracy Theories*, p. 129.

^
**16**
^
European Commission, *Rapid European SARS‐CoV‐2 Emergency Research Response*, Horizon, 2020–2023; https://cordis.europa.eu/project/id/101003589; L. W. Heyerdahl, et al., ‘Conditionality of COVID‐19 vaccine acceptance in European countries’, *Vaccine*, vol. 40, no. 9, 2022, pp. 1191–1197; https://www.sciencedirect.com/science/article/pii/S0264410X22000998?via%3Dihub (both accessed 11 April 2022).

^17^
A. Recio‐Román, M. Recio‐Menéndez, M. V. Román‐González, ‘Vaccine hesitancy and political populism. An invariant cross‐European perspective’, *International Journal of Environmental Research and Public Health*, vol. 18, no. 24, 2021; https://www.mdpi.com/1660-4601/18/24/12953 (accessed 11 April 2022).

^18^
J. Kennedy, ‘Populist politics and vaccine hesitancy in western Europe: an analysis of national‐level data’, *European Journal of Public Health*, vol. 29, no. 3, 2019, pp. 512–516, https://doi.org/10.1093/eurpub/ckz004 (accessed 11 April 2022).

^19^
D. Freeman, et. al., ‘Coronavirus conspiracy beliefs, mistrust and compliance with government guidelines in England’, *Psychological Medicine*, vol. 52, no. 2, 2022, pp. 251–263; https://www.cambridge.org/core/journals/psychological‐medicine/article/coronavirus‐conspiracy‐beliefs‐mistrust‐and‐compliance‐with‐government‐guidelines‐in‐england/9D6401B1E58F146C738971C197407461 (accessed 11 April 2022).

